# Clinical Risk Factors for High‐Dose Methotrexate‐Induced Oral Mucositis Following Individualized Dosing

**DOI:** 10.1002/cam4.70351

**Published:** 2024-11-01

**Authors:** Zhongbo Hu, Andrea M. Escalera‐Joy, Emily Ashcraft, Rushil Acharya, Sima Jeha, Cheng Cheng, Ching‐Hon Pui

**Affiliations:** ^1^ Hospitalist Medicine Program, Department of Oncology St. Jude Children's Research Hospital Memphis Tennessee USA; ^2^ Pediatric Oncology Education Program 2023, School of Medicine Ponce Health Sciences University Ponce Puerto Rico; ^3^ Department of Biostatistics St. Jude Children's Research Hospital Memphis Tennessee USA; ^4^ Department of Oncology St. Jude Children's Research Hospital Memphis Tennessee USA; ^5^ Department of Global Pediatric Medicine St. Jude Children's Research Hospital Memphis Tennessee USA; ^6^ Department of Pathology St. Jude Children's Research Hospital Memphis Tennessee USA

**Keywords:** acute kidney injury, acute lymphoblastic leukemia, delayed methotrexate clearance, high‐dose methotrexate, oral mucositis, risk factors

## Abstract

**Background:**

Oral mucositis affects about 20% of children undergoing high‐dose methotrexate (HDMTX) for acute lymphoblastic leukemia (ALL), despite existing management strategies. Personalized HDMTX dosing, adjusted by pharmacokinetics and leukemia risk, has reduced mucositis incidence, but variations still occur with similar 24‐h methotrexate levels.

**Methods:**

This retrospective study investigated risk factors for oral mucositis under individualized methotrexate protocols. Data from patients with ≥ Grade 2 oral mucositis (CTCAE v4.0) were analyzed from the St. Jude Children's Research Hospital total 16 trial. A 1:1 case–control matching method considered age, sex, risk classification, immunophenotype, and methotrexate course. McNemar's, Bowker's symmetry, and Wilcoxon signed‐rank tests were used for statistical analyses. Risk factors for recurrent mucositis were identified in a case‐only analysis.

**Results:**

The study found significant associations between methotrexate‐induced mucositis and new‐onset skin rashes (*p* = 0.0027), fever (*p* = 0.0016), neutropenic fever (*p* = 0.0008), lower absolute neutrophil count (*p* < 0.0001), acute kidney injury (AKI) (*p* = 0.0164), delayed methotrexate clearance (*p* = 0.0133), and higher 42‐h methotrexate levels (*p* = 0.0179). In the standard/high‐risk group, mercaptopurine dose was also linked to mucositis (*p* = 0.0495). Multivariable analysis showed that skin rashes (OR 6.5, *p* = 0.0016), fever (OR 2.8, *p* = 0.009), and neutropenia (OR 2.3, *p* = 0.0106) were independent risk factors for mucositis. Female sex (OR 7.12, *p* = 0.015) and AKI (OR 3.819, *p* = 0.037) were associated with recurrent mucositis.

**Conclusions:**

Fever, skin rashes, AKI, delayed methotrexate clearance, and higher 42‐h methotrexate levels were key risk factors for HDMTX‐induced oral mucositis. Skin rashes, fever, and neutropenia were independent predictors, while female sex and AKI were linked to recurrent mucositis.

## Graphical abstract

Rashes, fever, and neutropenia were independent risk factors for high‐dose methotrexate‐induced oral mucositis. Female sex and acute kidney injury were associated with recurrent mucositis.

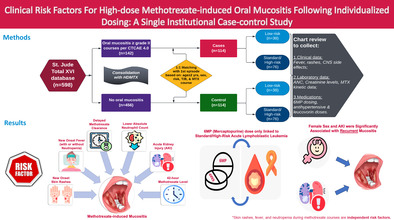

## 1 Introduction

High‐dose methotrexate (HDMTX) plays a critical role in overcoming MTX resistance, significantly reducing central nervous system (CNS) relapse and improving cure rates in childhood acute lymphoblastic leukemia (ALL) [1, 2, 3, 4, 5]. MTX depletes intracellular reduced folate by inhibiting thymidylate synthase and dihydrofolate reductase (DHFR), disrupting DNA and RNA synthesis and leading to cell death, particularly in high‐turnover tissues like the oral mucosa, resulting in oral mucositis [6, 7]. Despite careful MTX monitoring and supportive measures such as hydration, urine alkalinization, and leucovorin (LCV) rescue, approximately 20% of ALL patients undergoing HDMTX treatment experience oral mucositis (National Cancer Institute grade ≥ 3) [8, 9].

Since 1988, St. Jude Children's Research Hospital has pioneered individualized therapy based on pharmacokinetics [10]. The Total Therapy Study 12 introduced individualized MTX dose adjustments at 8‐h after initiating infusion (intracourse adjustment) to achieve target exposure levels (640–900 μM/h). This strategy increased five‐year continuous complete remission rates from 66% to 76% (*p* = 0.02) in patients with B‐ALL compared with fixed MTX doses of 1500 mg/m^2^ [10]. However, myelosuppression‐related toxicities and chemotherapy delays persisted, particularly in patients with high 42‐h MTX concentrations. Intensive hydration, alkalinization, and individualized LCV rescue mitigated life‐threatening toxicities, lowering high‐risk MTX concentrations (above 1 μM) from 22% to 7% of courses and mucositis incidence from 26% to 11% of courses [11].

Building on this, St. Jude Total Therapy Study 15 further refined individualized MTX dosing through intercourse adjustment based on clearance and risk classification. By targeting a steady‐state plasma concentration of 33 or 65 μM for low‐ or standard‐/high‐risk ALL patients respectively, the oral mucositis rate was decreased to 8.5% of the courses due to less delayed excretion [12].

Worldwide in 1998, a Danish study investigated the clinical and pharmacokinetic risk factors for HDMTX‐induced toxicity in children with ALL across 44 MTX courses [13]. The research reported that mucositis was the most common gastrointestinal side effect, typically developing by the third day after discharge and fully manifesting in all cases by day six. The incidence of oral mucositis (WHO grade ≥ 1) was approximately 52%. Several clinical and pharmacokinetic risk factors for HDMTX‐related mucositis were identified, including age, high plasma MTX concentration at 28 h, a low plasma 7‐hydroxymethotrexate (7‐OHMTX)/MTX ratio at 66 h, prolonged exposure to high plasma MTX concentrations (MTX area under the curve (AUC)), and slow MTX clearance [13].

Subsequent studies from different groups worldwide have investigated MTX‐associated oral mucositis from different perspectives. In 2014, a Dutch group reported that mucositis (NCI grade ≥ 3) was the most frequent toxicity during HDMTX (5 g/m^2^) phases, affecting 20% of 134 children with ALL (15% after the first course) [8]. They found that patients with mucositis had higher erythrocyte folate levels, though this was not associated with plasma MTX levels at 24 or 48 h, plasma folate, or plasma homocysteine levels. Other studies have identified various risk factors for HDMTX‐associated oral mucositis, such as the 7‐OHMTX/MTX ratio at 20 h [14], lower leukocyte counts, elevated liver function parameters [15], and the 48‐h MTX concentration [16]. However, these findings are not consistent due to varying conditions.

Genetic variations, particularly single nucleotide polymorphisms (SNPs) in MTX metabolic pathway genes, such as MTX efflux transporter gene ABCC4 and the organic anion transporter gene SLCO1B1, have been linked to inter‐individual differences in MTX toxicity [8, 17, 18]. Higher expression of the MTX influx transporter SLC19A1 has been correlated with favorable MTXPG accumulation in certain ALL subtypes, such as BCR‐ABL‐like and hyperdiploid ALL, while others exhibited lower SLC19A1 expression and reduced MTXPG intracellular accumulation [19].

Despite these advancements, the incidence of oral mucositis remains variable. This study aims to identify current clinical risk factors influencing oral mucositis incidence under contemporary protocols that individualize methotrexate dosing to maintain steady‐state plasma MTX concentrations during the first 24 h. This is the first retrospective study to systemically investigate the risk factors associated with oral mucositis in the largest cohort of pediatric patients with ALL, encompassing 2392 HDMTX courses, following individualized MTX dosage by intracourse and intercourse adjustments.

## 2 Method

### 2.1 Patients and Treatments

From October 29, 2007, to March 26, 2017, 598 eligible patients aged between 0.12 to 18.9 years with newly diagnosed ALL were enrolled in Total Therapy Study 16 at St. Jude Children's Research Hospital. Patients were classified as having low‐risk (LR), standard‐risk (intermediate‐risk), or high‐risk leukemia based on presenting characteristics and treatment response, determined by levels of minimal residual disease (MRD) measured by flow cytometry during remission induction and consolidation treatment.

After remission induction with prednisone, vincristine, daunorubicin, and PEG‐asparaginase, followed by cyclophosphamide, cytarabine, and thiopurine, patients received consolidation therapy consisting of four courses of HDMTX, mercaptopurine, and triple intrathecal therapy upon hematopoietic recovery. HDMTX was given based on the risk classification, with 2.5 g/m^2^ for LR group and 5.0 g/m^2^ for standard/high‐risk (SHR) group. Dosages were adjusted intracourse and intercourse based on pharmacokinetic data to achieve a steady‐state concentration of 33 μM in LR patients and 65 μM in SHR patients. LCV 15 mg/m^2^ (intravenous or orally) for SHR patients and 10 mg/m^2^(orally or intravenous) for LR patients was initiated 42 h after the start of methotrexate infusion and repeated every 6 h for a total of 3 doses, with adjustments based on plasma methotrexate levels. Patients with Down syndrome received 500 mg/m^2^ irrespective of risk group. Dasatinib was held 24 h before the administration and resumed when the methotrexate level dropped below the threshold for stopping LCV rescue. Then, all patients proceeded to antimetabolite‐based continuation therapy for 120 weeks.

### 2.2 MTX Concentration Monitoring

All patients had MTX concentrations measured prior to dosing and at 6, 23, and 42 h after the infusion began, using the ARK diagnostics homogeneous enzyme immunoassay (ARK Diagnostics', Fremont, CA) [12, 20]. If 42‐h MTX concentration exceeded 0.5 μM, levels were checked every 24 h until they dropped below 0.15 μM; otherwise, MTX level checks were discontinued. Patients showing any evidence of fluid collection or mucositis had plasma concentrations monitored until levels dropped detection limit (~0.06 μM by ARK assay). Significant changes in serum creatinine prompted continued monitoring of MTX concentrations even below 0.15 μM.

### 2.3 Data Recruitment

Patients' medical record numbers (MRNs) with oral mucositis grade 2 or above per Common Terminology Criteria for Adverse Events (CTCAE) Version 4.0 were retrieved from St. Jude Children's Research Hospital TOTAL 16 clinical trial study database. Clinical data, including demographic information (age, sex, ethnicity), ALL risk classification, immunophenotype (B‐cell or T‐cell), laboratory values (cytogenetics, molecular diagnosis, creatinine levels, absolute neutrophil counts (ANC), and MTX levels), concomitant medications, and adverse effect during the courses, were retrospectively reviewed for both cases and controls. Patients with Down syndrome were removed from the study due to significantly different MTX doses.

### 2.4 Definitions

A case was defined as the first occurrence of oral mucositis grade 2 or higher during consolidation treatment. Acute Kidney Injury (AKI) was defined as an increase in serum creatinine by ≥ 0.3 mg/dL from baseline (≥ 26.5 μmol/L) within 48 h; or an increase in serum creatinine to ≥ 1.5 times baseline within the previous 7 days. A MTX course was defined from the time start of MTX infusion to the time before the next cycle of MTX begins (typically 2 weeks) or until the patient returned to baseline prior to the next chemotherapy cycle. Delayed methotrexate elimination (clearance) was defined as a concentration above 1 μM after 42 h, above 0.15 μM after 66 h, or above 0.05 μM after 96 h from the start of infusion [21]. MTX area under the curve (AUC) values were calculated by the free online tool generated by MTXPK.org [22, 23]. The definitions of the ploidy levels followed the “International System of Cytogenetic Nomenclature”: hypodiploid (35–45 chromosomes) and hyperdiploid (47–58 chromosomes) [24]. In TOTAL 16 study, high hyperdiploid was further classified as more than 50 chromosomes or DNA index ≥ 1.16.

### 2.5 Study Design and Statistics

A 1:1 matched case–control study was used to identify differences in risk factors that may be associated with oral mucositis between patients who exhibited at least one mucositis event during consolidation (case) and patients without mucositis during consolidation (control). Matching criteria included: MTX course (1–4), final risk (LR vs. SHR), age (within 2 years), immunophenotype (B or T cell), and sex. Comparisons between cases and controls were stratified by final risk because SHR group's targeted plasma 24‐h MTX levels were nearly double those of the LR group (65 μM vs. 33 μM). Recurrent mucositis‐case only analysis was performed to determine differences between patients with one mucositis versus those with 2 or more.

Statistical comparisons between cases and controls stratified by risk group utilized McNemar's test or Bowker's symmetry test for categorical variables and Wilcoxon signed‐rank test for continuous variables. In the cases‐only analysis, comparisons used the chi‐square test or exact chi‐square test for categorical variables and the Wilcoxon rank sum test for continuous variables. Categorical variables are displayed as *N*% and continuous variables are displayed as median (minimum‐maximum) unless specifically mentioned.

Multiple logistic regression models were created to better assess prognostic value of risk factors on mucositis. Characteristics that were statistically significant at < 0.1 level in univariable analyses were considered for multiple logistic regression models. Odds ratios, 95% Wald confidence intervals, and *p*‐values are reported for analyses among cases and controls and cases only. An alpha of 0.05 was considered statistically significant. All analysis and case–control matching were conducted using SAS 9.4 (Cary, NC).

## 3 Result

### 3.1 Patient Characteristics

In total, about 19.06% of patients had mucositis during consolidation with HDMTX (CTCAE grade 2 or above, 114 out of 598 patients). 5.7% of the MTX courses (136 out of 2392) were complicated with oral mucositis (CTCAE grade 2 or above).

### 3.2 Analyses From the Case–Control Matching

**FIGURE 1:**
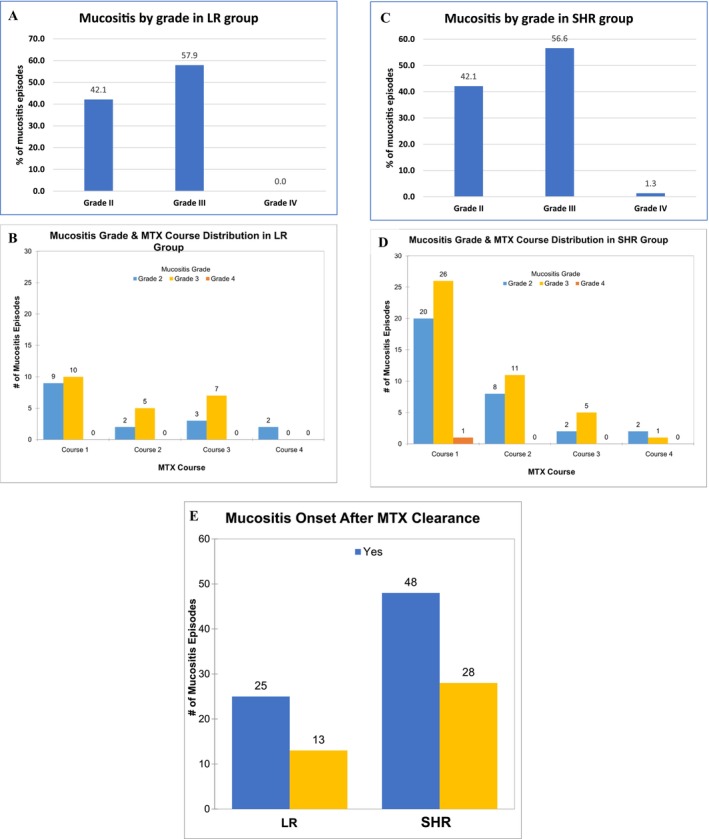
The distribution of mucositis episodes. (A) The distribution of mucositis by grade during MTX courses in LR group. (B) The distribution of mucositis in detail by grade and MTX courses in LR group. (C) The distribution of mucositis by grade during MTX courses in SHR group. (D) The distribution of mucositis in detail by grade and MTX courses in SHR group. (E) The relationship between mucositis onset and MTX clearance. Yes, means mucositis onset after MTX clearance. No, means mucositis onset before MTX clearance. MTX, methotrexate. LR, low risk. SHR, stand/high risk.

The case–control model included 228 cases and controls (Table 1). 36.8% of LR patients (total 38) and 48.7% of SHR patients (total 76) were females. Most patients (97.4% of LR, 50.0% of SHR) were aged 1–10. In the mucositis case groups, 50.0% in the LR group and 61.8% in the SHR group had mucositis in course 1, 18.4% (LR) and 25.0%(SHR) in course 2, 26.3% (LR) and 9.2% (SHR) in course 3, and 5.3% (LR) and 3.9% (SHR) in course 4. The severity of mucositis was grade 2 in 42.1% of the LR group and 42.1% of the SHR; grade 3 in 57.9% of the LR and 56.6% of the SHR group; and grade 4 in 0.0% of the LR and 1.3% of the SHR group (Figure 1A,C). The detailed distribution of patient numbers is also shown (Figure 1B,D). Mucositis onset occurred after MTX was cleared in about 65.8% (25 out of 38) of the LR group and 63.2% (48 out of 76) of the SHR group (Figure 1E).

**TABLE 1:** Clinical features of the patients with the first episode of mucositis (case) comparing with matched controls (control) during HDMTX.

Characteristics	LR patients		SHR patients	
	Case (*N* = 38)	Control (*N* = 38)	Case (*N* = 76)	Control (*N* = 76)
Age, years				
< 1	0	0	3 (3.9)	2 (2.6)
1–10	37 (97.4)	37 (94.9)	38 (50.0)	36 (47.4)
> 10	1 (2.6)	1 (2.6)	35 (46.1)	38 (50.0)
Sex				
Male	24 (63.2)	24 (63.2)	39 (51.3)	39 (51.3)
Female	14 (36.8)	15 (36.8)	37 (48.7)	37 (48.7)
Race				
White	27 (71.1)	30 (78.9)	51 (67.1)	56 (73.7)
Black	7 (18.4)	6 (15.8)	19 (25.0)	15 (19.7)
Other	4 (10.5)	2 (5.3)	6 (7.9)	5 (6.6)
Immunophenotype				
B‐cell	38 (100)	38 (100)	56 (73.7)	56 (73.7)
T‐cell	0	0	20 (26.3)	20 (26.3)
HDMTX course				
1	19 (50.0)	19 (50.0)	47 (61.8)	47 (61.8)
2	7 (18.4)	7 (18.4)	19 (25.0)	19 (25.0)
3	10 (26.3)	10 (26.3)	7 (9.2)	7 (9.2)
4	2 (5.3)	2 (5.3)	3 (3.9)	3 (3.9)
Oral mucositis grade				
2	16 (42.1)	NA	32 (42.1)	NA
3	22 (57.9)	NA	44 (56.6)	NA
4	0 (0)	NA	1 (1.3)	NA
Mucositis onset after MTX clearance				
Yes	25 (65.8)	NA	48 (63.2)	NA
No	13 (34.2)	NA	28 (36.8)	NA

*Note:* () shows in % inside the content of the table.

Abbreviations: LR, low risk; NA, none available; SHR, standard/high risk.

### 3.3 Clinical Presentation Effect

**FIGURE 2:**
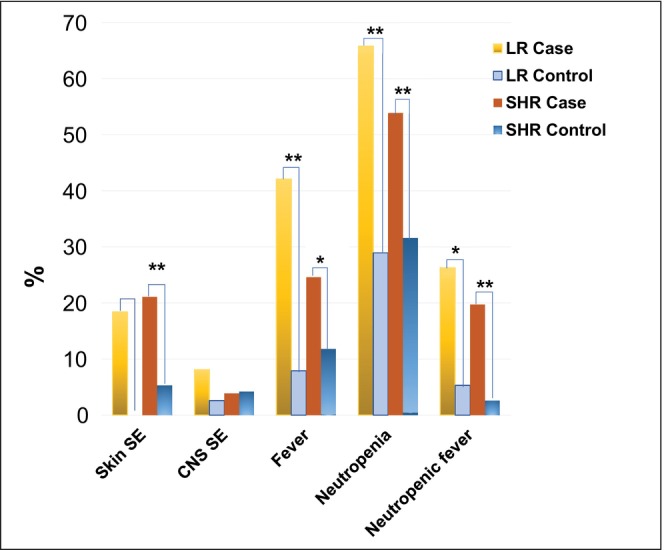
Clinical symptoms during HDMTX such as new onset skin rashes, CNS side effects, fever, neutropenia, and neutropenic fever that might be associated with oral mucositis. *Note:* * *p* < 0.05, ** *p* < 0.01.

New‐onset skin rashes after HDMTX occurred in 18.4% of LR cases and 21.1% of SHR cases, significantly higher compared to controls (0 in LR group; 5.3% SHR, *p* = 0.0027) (Table S1 and Figure 2). CNS side effects such as headache, seizure, stroke‐like symptoms, speech disturbances, or encephalopathy developed in 8.1% of LR cases and 3.9% of SHR cases, with no significant differences from controls (LR: 2.6%, *p* = 0.3173; SHR: 4.2%, *p* = 1). New‐onset fever (temperature above 38°C) occurred in 42.1% of LR cases and 25.0% of SHR cases, significantly higher compared to controls (LR: 7.9%, *p* = 0.0016; SHR: 11.8%, *p* = 0.0124). Neutropenic fever (fever with ANC below 500/mm^3^) developed in 26.3% of LR cases and 19.7% of SHR cases, also significantly higher compared to controls (LR: 5.3%, *p* = 0.0209; SHR: 2.6%, *p* = 0.0008) (Table S1 and Figure 2).

### 3.4 Laboratory Effect

**FIGURE 3:**
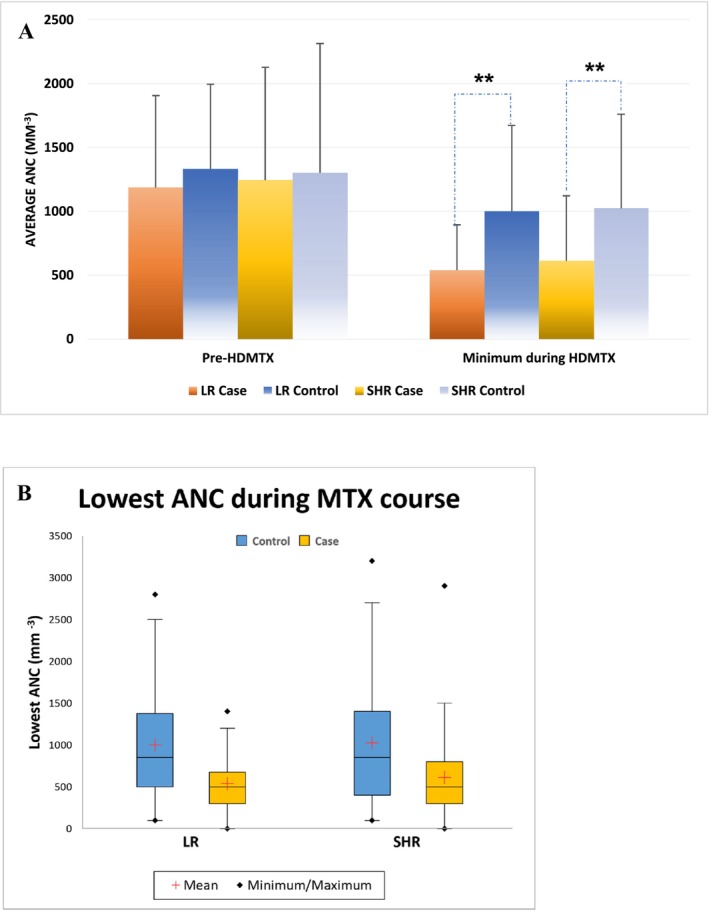
The effect of absolute neutrophil counts (ANC) before and after HDMTX on the MTX‐associated mucositis. (A) Box plot shows the baseline ANC before MTX was started and the lowest ANC during the MTX course. (B) Bar charts show the ANC before the MTX initial and during MTX course. *Note:* * *p* < 0.05, ** *p* < 0.01.

The criteria to start HDMTX course were an ANC ≥ 300/mm^3^ and white blood cells ≥ 1000/mm^3^. We aimed to assess the effects of baseline ANC and neutrophil counts after MTX initiation on the development of oral mucositis. Because the protocol set up the criteria of ANC to start HDMTX, we did not find differences in ANC before starting HDMTX between LR cases (1186.4 ± 719.6 per mm^3^) or SHR group (1246.1 ± 880.7 per mm^3^) compared to controls in (LR: 1334.7 ± 660.4 per mm^3^, *p* = 0.2844; SHR: 1303.2 ± 1009.9 per mm^3^, *p* = 0.9362) (Table S2 and Figure 3A **)**. New onset neutropenia (ANC less than 500/mm^3^) during the MTX course occurred in 65.8% of LR cases and 53.9% of SHR cases, compared to controls (LR: 28.9%, *p* = 0.006; SHR: 31.6%, *p* = 0.0079) (Figure 2). The lowest ANC during HDMTX was significantly lower in LR cases (540.5 ± 354.5 per mm^3^) and SHR cases (613.8 ± 508.0 per mm^3^) compared to controls (LR: 1000.8 ± 671.6 per mm^3^, *p* = 0.0007; SHR: 1025.0 ± 735.8 per mm^3^, *p* < 0.0001) (Table S2 and Figure 3B).

**FIGURE 4:**
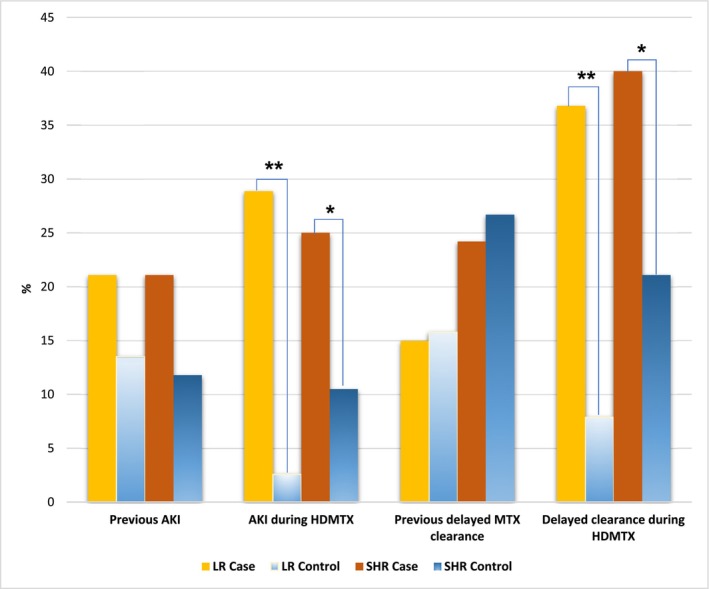
The effect of acute kidney injury and delayed methotrexate (MTX) clearance previously or during the high‐dose MTX (HDMTX) course on the oral mucositis. *Note:* * *p* < 0.05, ** *p* < 0.01.

Out of 38 LR cases, 8 (21.1%) had a previous history of AKI, which was not significantly different compared to the controls (13.5%, *p* = 0.3657). Similarly, in SHR group, 16 out of 76 cases (21.1%) had a history of AKI before HDMTX treatment started, compared to the control group (11.8%, *p* = 0.1444). During the HDMTX courses, AKI occurred in 11 out of 38 cases (28.9%) in the LR group and in 19 out of 76 cases (25%) in SHR group, which were both significantly higher than their respective controls (2.6% in LR, *p* = 0.00039; 10.5% in SHR, *p* = 0.0164). Previous delayed MTX clearance was observed in 15% of LR cases and 24.2% of SHR cases, which were not significantly different from the controls (15.8% of LR, *p* = 1; 26.7% of SHR, *p* = 0.7389). However, delayed MTX clearance during the HDMTX courses happened in 14 out of 38 cases (36.8%) in LR group and in 30 out 76 cases (40%) in SHR group, which were significantly higher compared to their controls (7.9% in LR group, *p* = 0.0045; 21.1% in SHR group, *p* = 0.0133) (Table S2 and Figure 4).

### 3.5 MTX Kinetics Effect

**FIGURE 5:**
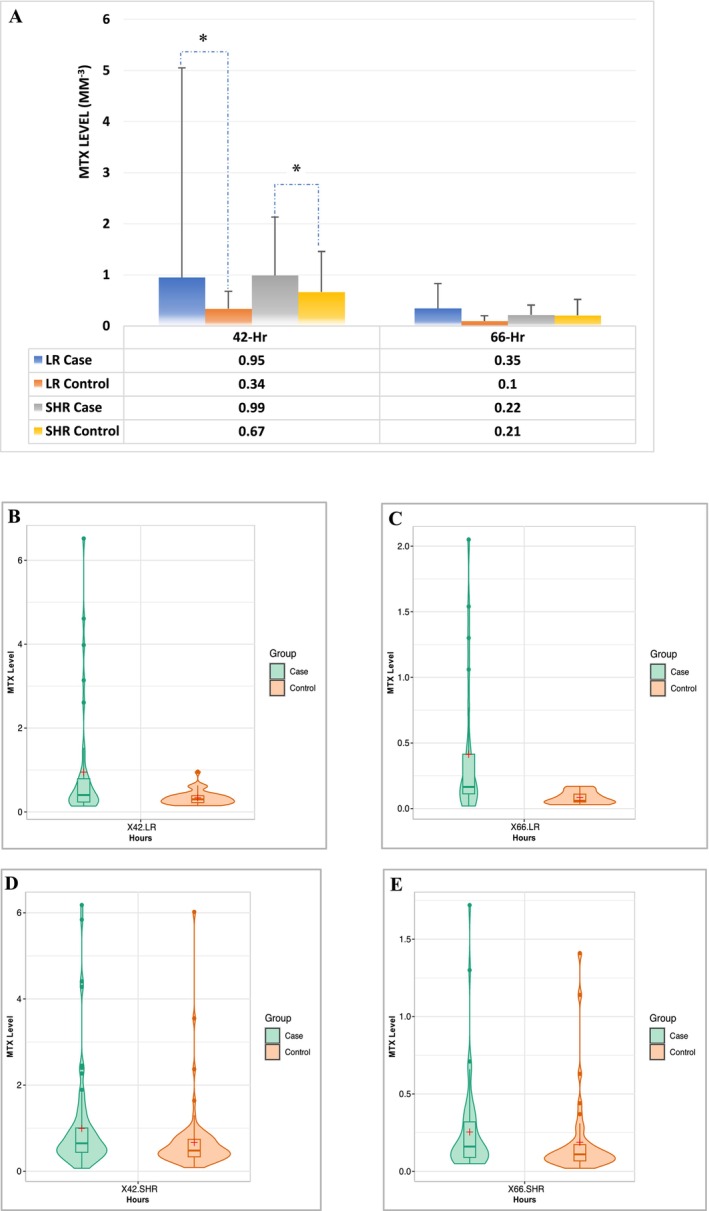
MTX kinetics that might be associated with oral mucositis during HDMTX. Only 42‐h MTX level is associated with HDMTX‐induced oral mucositis in both LR and SHR groups. But 66‐h MTX level is not. *, *p* < 0.05 compared with control. (A) MTX levels at 42 h and 66 h compared with the controls. (B, C) Violin plot shows the MTX levels at 42 h (B) and 66 h (C) compared with the controls (*n* = 38) in LR group. (D, E) Violin plot shows the MTX levels at 42 h (D) and 66 h (E) compared with the controls (*n* = 38) in SHR group. (B–E) the box plots inside the violin show 25 percentiles, medians, and 75 percentiles; + shows the mean.

MTX dosages were similar between case and control groups (each 38 patients) in both LR (2479.0 ± 129.8 mg/m^2^ vs. 2426.0 ± 251.9 mg/m^2^, *p* = 0.3125) and SHR groups (each 76 patients) (4662.1 ± 728.9 mg/m^2^ vs. 4671.2 ± 645.0 mg/m^2^, *p* = 0.9055) (Table S3 and Figure S1A). MTX AUC was not different in LR group (937.1 ± 330.1 μM × h. vs. 810.4 ± 190.2 μM × h., *p* = 0.1035) or SHR group (1869.0 ± 466.1 μM × h. vs. 1745.4 ± 418.3 μM × h., *p* = 0.8920) compared to controls (Table S3 and Figure S1B). However, 42‐hour MTX levels were associated with HDMTX‐induced oral mucositis in both LR (0.95 ± 4.10 μM vs. 0.34 ± 0.17 μM, *p* = 0.0115) and SHR group (0.99 ± 1.14 μM vs. 0.67 ± 0.79 μM, *p* = 0.0179) compared to controls. 66‐Hr MTX levels were not different in LR group (0.35 ± 0.48 μM vs. 0.10 ± 0.05 μM, *p* = 0.2617) or SHR group (0.22 ± 0.19 μM vs. 0.21 ± 0.31 μM, *p* = 0.2809) compared to controls (Figure 5A). The distribution of the MTX levels in LR group was wider in cases vs. control both at 42 h (Min‐Max: 0.14–6.52 vs. 0.15–0.96, Median: 0.41 vs. 0.30), and at 66 h (Min‐Max: 0.04–1.30 vs. 0.03–0.17, Median: 0.13 vs. 0.10) indicating the MTX metabolic variation (Figure 5B,C). The distribution of the MTX levels in SHR group was similar in cases as in controls both at 42 h (Min‐Max: 0.07–6.18 vs. 0.09–6.02, Median: 0.65 vs. 0.48), and at 66 h (Min‐Max: 0.05–0.71 vs. 0.02–1.41, Median: 0.13 vs. 0.11) (Figure 5D,E).

Of note, accumulated LCV rescue dosages were significantly higher both in LR cases (210.9 vs. 34.6 mg/m^2^, *p* < 0.001) and SHR cases (238.8 vs. 81.3 mg/m^2^, *p* < 0.0001) compared to controls. (Table S3 and Figure S1C).

### 3.6 Medication Effect

**FIGURE 6:**
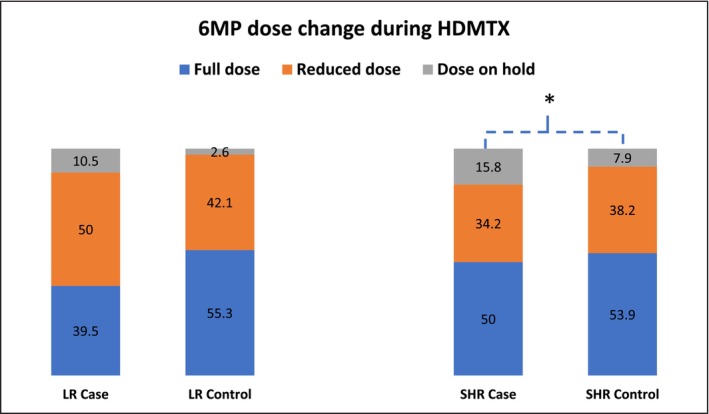
6MP dose changes during HDMTX that affect oral mucositis. Number is shown in % inside the content of the table. *Note:* * *p* < 0.05, ** *p* < 0.01.

Concurrent medications, such as NSAIDS and mercaptopurine (6MP), were analyzed. None of the patients in the case nor control group used NSAIDS. About half of the patients in the study group had reduced 6MP dose. In the LR group, 6MP doses in about 10.5% of cases were on hold, with no statistical difference compared to controls. In the SHR group, 34.2% of the cases had reduced 6MP doses and 15.8% cases 6MP on hold, with the latter rate significantly higher than 7.9% of the control group (*p* = 0.0495) (Figure 6 and Table S4). There were no differences in anti‐hypertensive medicine or TMP/SMX doses (Table S4).

### 3.7 Cytogenetics and Molecular Diagnosis Effect

Intracellular accumulation of the active form of MTX metabolite‐methotrexate polyglutamates has been found to be highly correlated with some molecular or cytogenetic type of ALL, such as hyperdiploid and BCR::ABL‐like ALL. However, ETV6::RUNX1, TCF3::PBX1, and T‐ALL had low MTXPG intracellular accumulation in leukemia cells [19]. Our analysis did not find a significant relationship between typical molecular diagnoses, such as KMT2A, BCR::ABL1, and ETV6::RUNX1 rearrangement and oral mucositis, nor the ploidy levels or immunophenotype (Table S5).

### 3.8 Logistic Regression Analysis

Multivariable analyses showed that skin rashes (odds ratio (OR) 6.5, 95% CI: 2.024–20.707, *p* = 0.0016), fever (OR 2.8, 95% CI: 1.298–6.205, *p* = 0.009), and neutropenia (OR 2.3, 95% CI: 1.211–4.252, *p* = 0.0106) during the MTX courses were independent factors and the patients who had one of these factors were more likely to have mucositis. Delayed MTX clearance during the course had a moderate likelihood but was not statistically significant (OR 2.2, 95% CI: 0.973–5.171, *p* = 0.0582). The 42‐h MTX level was not significantly associated with mucositis (OR 1.2, 95% CI: 0.733–1.957, *p* = 0.4709) (Table 2).

**TABLE 2:** Multivariable analysis for odds of mucositis with risk factors and baseline patient characteristics.

Odds of mucositis controlling for risk factors and baseline characteristics response: case versus control					
Effect		Odds ratio	95% Wald confidence limits		*p*
Final risk	Standard/high versus low	0.907	0.477	1.724	0.7650
Skin SE	Yes versus no	6.475	2.024	20.707	**0.0016**
AKI current course	Yes versus no	1.883	0.724	4.902	0.1945
Delayed MTX clearance current course	Yes versus no	2.243	0.973	5.171	0.0582
6MP dose	Reduced/hold versus full	1.199	0.639	2.250	0.5712
Fever	Yes versus no	2.838	1.298	6.205	**0.0090**
Neutropenia	Yes versus no	2.269	1.211	4.252	**0.0106**
MTX 42 h level		1.198	0.733	1.957	0.4709

Abbreviations: 6MP, mercaptopurine; AKI, acute kidney injury; MTX, methotrexate; SE, side effects. *Note:* Bold values signifies the *p* < 0.05.

### 3.9 Case‐Only VS Recurrent Mucositis Analysis

**FIGURE 7:**
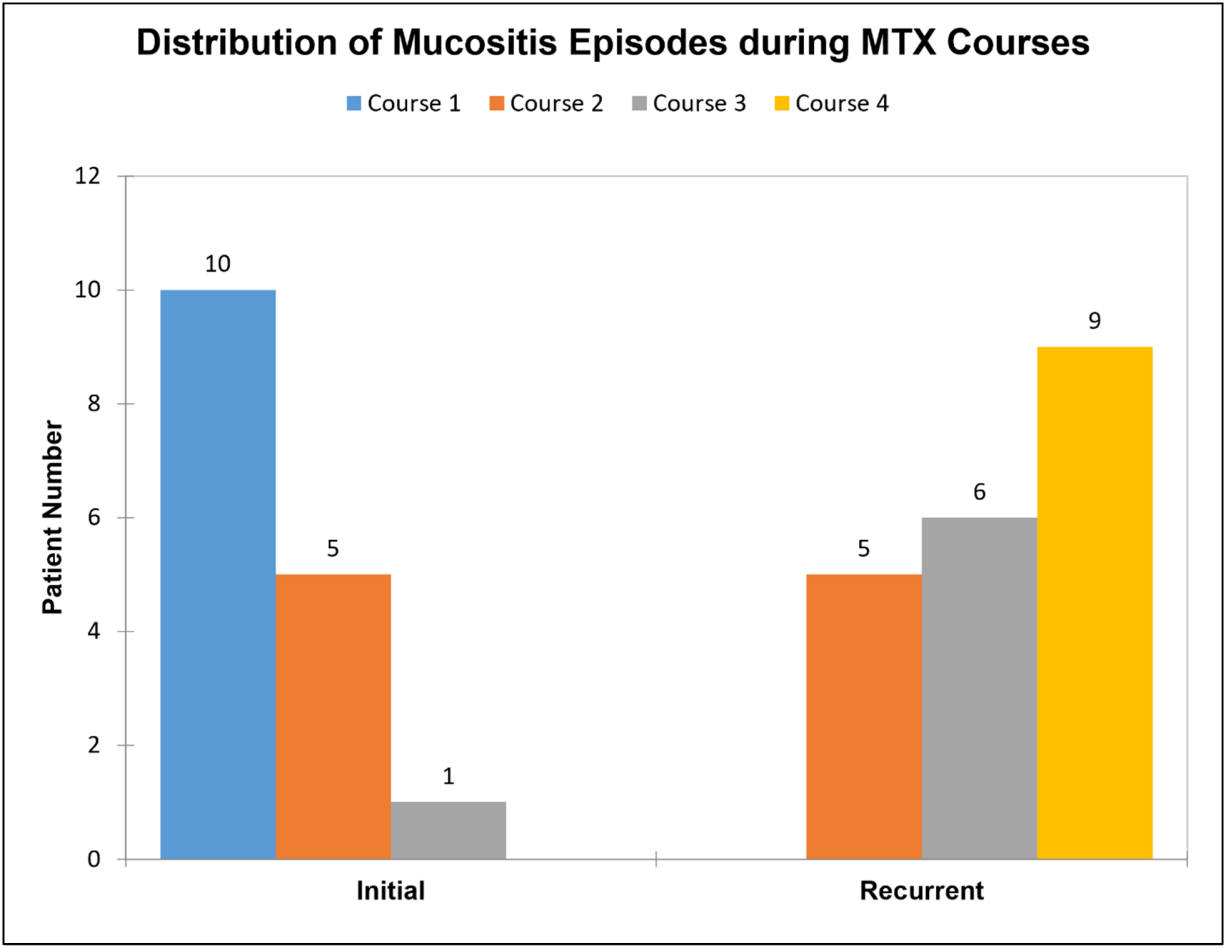
The patient number in all the patients with recurrent mucositis episodes, comparing initial HDMTX courses with the recurrent courses.

The case‐only analysis included 114 cases, with one case removed due to missing data. A total of 16 patients experienced recurrent mucositis across 20 courses. Two patients had mucositis in all four HDMTX courses, while the others had only one recurrent episode. The distribution of recurrent episodes was 5 cases in course 2, 6 in course 3, and 9 in course 4 (Figure 7 **)**. Recurrent mucositis was significantly associated with female sex (75%, *p* = 0.0086) and AKI (56.3%, *p* = 0.0158) (Table 3). The ANC and MTX kinetics parameters were not significantly associated with recurrent mucositis. However, accumulated LCV doses were significantly associated with recurrent mucositis (*p* = 0.017) (Table S6). Multivariable logistic regression analysis showed that females were 7.1 times more likely to have recurrent mucositis (OR 7.12, 95% CI: 7.582–32.034, *p* = 0.0105), and patients with AKI before or during the MTX course at any time were more likely to have recurrent mucositis (OR 3.819, 95% CI: 1.084–13.45, *p* = 0.037) (Table 4). Although not statistically significant when controlling for other covariates, patients with CNS side effects and those with a history of delayed MTX clearance had a higher chance to develop recurrent mucositis, albeit lacking alpha = 5% statistical significance (OR 8.839, 95% CI: 0.865–90.275, *p* = 0.0661 and OR 3.177, 95% CI: 0.813–12.416, *p* = 0.0964).

**TABLE 3:** Univariable analysis for the correlation of clinical and laboratory features associated with recurrent oral mucositis.

Differences in clinical features by number of mucositis toxicities			
Clinical feature	1 versus 2+ mucositis toxicities		
	1 mucositis toxicity (*n* = 98)	2+ mucositis toxicities (*n* = 16)	*p*
Age			
< 1	3 (3.1)	0 (0.0)	0.1025
1–9 years	68 (69.4)	7 (43.8)	
≥ 10	27 (27.6)	9 (56.3)	
Sex			
Male	59 (60.2)	4 (25.0)	0.0086*
Female	39 (39.8)	12 (75.0)	
Race			
White	68 (69.4)	10 (62.5)	0.203
Black	20 (20.4)	6 (37.5)	
Other	10 (10.2)	0 (0.0)	
Final risk			
Low	36 (36.7)	2 (12.5)	0.0843
Standard/high	62 (63.3)	14 (87.5)	
Subtype			
KMT2A	7 (8.5)	0 (0.0)	0.349
Ph+	2 (2.4)	1 (8.3)	
ETV6::RUNX1	23 (28.0)	1 (8.3)	
Hyperdiploid	19 (23.2)	4 (33.3)	
Low hypodiploid	50 (61.0)	10 (83.3)	
Other	1 (1.2)	0 (0.0)	
Immunophenotype			
T‐ALL	16 (16.3)	4 (25.0)	0.477
B‐ALL	82 (83.7)	12 (75.0)	
Mucositis after clearance (any course)			
Yes	65 (66.3)	14 (87.5)	0.142
No	33 (33.7)	2 (12.5)	
Ever had skin SE			
Yes	20 (20.4)	5 (31.3)	0.3388
No	78 (79.6)	11 (68.8)	
Ever had CNS SE			
Yes	4 (4.1)	3 (18.8)	0.0564
No	94 (95.9)	13 (81.3)	
Ever had AKI			
Yes	24 (24.5)	9 (56.3)	**0.0158**
No	74 (75.5)	7 (43.8)	
History of delayed clearance in any course			
Yes	44 (44.9)	11 (68.8)	0.0767
No	54 (55.1)	5 (31.2)	
Ever received hypertension meds			
Yes	4 (4.1)	3 (18.8)	0.0564
No	94 (95.9)	13 (81.3)	
Ever had fever			
Yes	30 (30.6)	7 (43.8)	0.2980
No	68 (69.4)	9 (56.3)	
Ever had neutropenia			
Yes	60 (61.2)	12 (75.0)	0.2895
No	38 (38.8)	4 (25.0)	
Ever had neutropenic fever			
Yes	23 (23.5)	6 (37.5)	0.2323
No	75 (76.5)	10 (62.5)	

*Note:* () shows in % inside the content of the table. Bold values signifies the *p* < 0.05. * indicated significance during case‐control assay.

**TABLE 4:** Multivariable logistic analysis for odds of recurrent mucositis controlling for risk factors.

Odds of recurrent mucositis controlling for risk factors and baseline characteristics. Response: 2+ toxicities versus 1 toxicity					
Effect		Odds ratio	95% Wald confidence limits		*p*
Final risk	Standard/high versus low	3.72	0.67	20.651	0.1331
Sex	Female versus male	7.12	1.582	32.034	**0.0105**
Any CNS SE	Yes versus no	8.839	0.865	90.275	0.0661
Any AKI	Yes versus no	3.819	1.084	13.45	**0.037**
Any hypertension meds	Yes versus no	5.923	0.674	52.086	0.1088
History of delayed clearance in any course	Yes versus no	3.177	0.813	12.416	0.0964

*Note:* Bold values signifies the *p* < 0.05.

## 4 Discussion

HDMTX has been used to treat various cancers, including ALL, lymphoma, osteosarcoma, medulloblastoma, and some adult solid tumors [1]. Despite advancements in individualized dosing, pharmacogenetic studies, and proper measures including MTX level monitoring, hyperhydration, urine alkylation, avoidance of medications that might affect MTX clearance, and LCV rescue, oral mucositis remains a common side effect. In the St Jude Total studies, individualized dosing reduced oral mucositis above CTCAE grade 2 to 5.7% of MTX courses, one of the lowest reported rates [8].

To identify the risk factors influencing oral mucositis incidence under contemporary individualizing MTX dosing protocols, we performed two analyses: a 1:1 matching case–control study and a case‐only analysis. Each patient received four courses of high‐dose MTX. Patients who experienced a single episode of mucositis may have different clinical or biological features compared to those who developed multiple episodes of mucositis. To minimize the confounding effects of different MTX courses, we first performed the matched case–control study. This study confirmed that skin rashes, fever, febrile neutropenia, lower ANC, AKI, and delayed MTX clearance during the course, and 42‐h MTX levels are significantly associated with MTX‐induced mucositis. To further explore why 16 patients developed recurrent mucositis, we conducted the case‐only study. This analysis revealed that female sex and AKI were significantly associated with recurrent mucositis.

MTX levels, particularly those exceeding 1 μmol/L at 42 h, have been significantly associated with oral mucositis in early studies [11, 13]. Higher MTX levels at various time points are linked to delayed MTX clearance [13, 25]. Historical studies suggest that delayed MTX clearance is the best surrogate marker for HDMTX‐associated toxicity [26]. In our study, we first conducted univariable analyses to assess the association between the variables of interest and mucositis. Multivariable analyses were then performed to determine whether the factors found significant in the univariate analyses (*p* ≤ 0.1) independently affected the outcome when controlling for other factors.

Our study confirmed that 42‐h MTX level, AKI, and delayed MTX clearance during MTX course were associated with the incidence of mucositis in the univariable analysis. However, in the multivariable analysis, MTX levels, delayed MTX clearance, and AKI were no longer independently associated with mucositis, indicating that these factors may not be independent predictors for oral mucositis. These factors could be correlated with each other or with other factors. Specially, we observed that only 19 patients with delayed MTX clearance subsequently developed AKI during the MTX course, while 34 patients with delayed MTX clearance did not develop AKIs. Pharmacogenomic factors, such as SNPs in MTX efflux transporter genes ABCB1 [27] and ABCC4 [8], the organic anion transporter gene SLCO1B1 [17, 18], the MTX influx transporter SLC19A1 [28], and MTX metabolism enzymes like 5,10‐methylenetetrahydrofolate reductase (MTHFR) [27], may influence MTX clearance and serve as risk predictors for MTX‐induced mucositis. This finding underscores the importance of individualizing treatment through intercourse and intracourse adjustments based on MTX kinetics and pharmacogenomics, minimizing the impact of genetic variations on treatment outcomes.

AKI, which delays the MTX clearance, was a key risk factor for recurrent mucositis. Previous AKI indicated baseline damage of renal tubule, which makes the patient susceptible for further injury during MTX course [29]. AKI developed during the MTX course prolongs the exposure to MTX and increases the chance of mucositis even after dose individualization. AKI during the MTX course might be the early sign of MTX‐induced renal damage, including crystal nephritis and direct tubular damage [30].

Our multivariable analyses showed that fever and neutropenia during MTX courses were independent factors for mucositis. Neutrophils, as the first line of defense, play a crucial role in protection against infections and promote wound healing. A decreased neutrophil count can be caused by MTX‐induced bone marrow suppression after prolonged exposure or other factors. Earlier Studies have demonstrated the relationship between oral mucositis, fever, and neutropenia [31, 32]. Fever is a symptom of systemic inflammatory response. MTX induces DNA damage and causes the release of reactive oxygen species, leading to epithelial cell death. Additionally, immune cells, mainly macrophages, can be activated through pattern recognition receptors binding to pathogen‐related components or components released from damaged cells, leading to the assembly of the inflammasomes [31]. This activates the inflammatory process, resulting in the release of inflammatory cytokines such as TNF‐α, IL‐1β, and IL‐6, interferon‐γ, which cause fever. Therefore, fever could be a consequence of mucositis, or systemic inflammation.

Dermatologic toxicity during HDMTX can range from mild transient erythematous eruptions to frank exfoliative dermatitis, affecting up to 10%–15% of patients, typically presenting as nonspecific morbilliform drug rashes [1]. Our case–control study identified skin rashes as a significant independent factor for oral mucositis. The mechanism of MTX‐induced dermatologic toxicity may be similar to that of MTX‐induced mucositis as dermatitis might reflect the prolonged tissue exposure from MTX and serve as an early sign of mucositis.

Another significant finding of our retrospective case study is that female sex was identified as a risk factor for recurrent mucositis. This has been reported in several studies related to chemotherapy‐induced mucositis, including MTX or transplant‐associated cases [33, 34, 35]. It is known that sex hormones influence the physiology of the oral cavity. High‐dose progesterone, for instance, has been shown to reduce the antibacterial activity of neutrophils [36]. Hormonal changes in female during chemotherapy and transplant might play a fundamental role in the etiopathogenesis of chemotherapy‐induced mucositis [35]. Further mechanism studies are needed to confirm this effect.

Since methotrexate is primarily cleared through renal excretion, patients with underlying renal dysfunction are at an increased risk of having delayed clearance. The elevated and prolonged exposure to methotrexate resulting from delayed clearance increases susceptibility to complications such as mucositis and myelosuppression [37]. Many medications inhibit renal excretion of MTX and increase treatment‐related toxicity. Well‐studied drugs include nonsteroidal anti‐inflammatory drugs (NSAIDs), phenytoin, ciprofloxacin, penicillin‐type drugs, probenecid, amiodaron, and proton pump inhibitors. Sulfa drugs, such as trimethoprim‐sulfamethoxazole (TMP/SMX, Bactrim), can also inhibit MTX renal excretion. Trimethoprim is reported to compete with MTX for binding sites on DHFR, molecules. Significant drug–drug interactions between TMP/SMX and MTX have been documented [38]. However, recent studies have confirmed that prophylactic sulfamethoxazole/trimethoprim has minimal effect on the pharmacokinetics or pharmacodynamics of MTX and does not cause delayed clearance or increased toxicities [39]. Therefore, it can be safely used concurrently with HDMTX [39, 40]. Most of our patients (data not shown, 9 out of 136 cases and 12 out of 136 controls received pentamidine instead of Bactrim) were on Bactrim and did not show any significant differences in the incidence of mucositis.

MTX and 6‐mercaptopurine (6‐MP) are commonly used together in the treatment of childhood ALL. Our study found that a higher dose of 6MP combined with a higher MTX dose in standard‐/high‐risk group significantly increased the incidence of mucositis. Several studies indicate a synergistic effect between MTX and 6‐MP, although MTX may increase the bioavailability of 6‐MP [41]. Lowering the dose of 6‐MP co‐administered during chemotherapy in childhood ALL could be a strategy to reduce the risk of severe bone marrow toxicity following high‐dose MTX.

The accumulated LCV doses were highly associated with oral mucositis, not as a risk factor but as an outcome of mucositis and related factors. The LCV dosages were primarily influenced by MTX concentration, while the patient's serum creatinine levels, previous toxicities, and clinical symptoms of mucositis also affected the frequency and dosing of LCV. High cumulative LCV doses and early initiation of LCV after the start of MTX can help reduce the oral mucositis [42].

Other factors that we did not investigate include bilirubin levels, which have been shown to be associated with a higher prevalence of mucositis [34]. Additionally, nutritional status, poor dental hygiene, and previous exposure of immunosuppressive therapy, all of which significantly affect oral mucositis, were not examined in our study. Although our research included the largest cohort of pediatric patients with ALL, encompassing 2392 HDMTX courses in 578 patients, there are still limitations in the patient population and sample size. As a result, the interpretation of our findings may differ from those of other pediatric series. Furthermore, the retrospective nature of the study only indicates association rather than establishment the cause‐and‐effect relationships.

Our study emphasizes the importance of identifying risk factors for mucositis to enable early intervention. While planning HDMTX treatment or during the HDMTX course, the emergence of risk factors such as new‐onset changes, fever or neutropenic fever, lower ANC, AKI or delayed MTX clearance during the MTX course, or high 42‐h methotrexate level (> 1 μM) should prompt consideration of extensive preventive strategies. These strategies may include increasing LCV dosing, continuing hyperhydration, initiating early phototherapy (also called photobiomodulation, or low‐level laser therapy) [43], and using glutamine [44, 45] or keratinocyte growth factor (palifermin) [46] to help prevent or mitigate mucositis.

For patients with significant AKI or delayed MTX clearance, the risk of MTX‐associated toxicity, including oral mucositis, is markedly increased. The literature indicates that when plasma MTX concentration exceeds 10 μM for more than 36 or 42 h, or when serum creatinine is more than 1.5 times the upper limit of normal and the plasma MTX concentration is ≥ 2 standard deviation above the mean ≥ 12 h after MTX administration, glucarpidase has been effective in treating HDMTX‐induced renal dysfunction and reducing the incidence of associated toxicities, including mucositis [47, 48]. The optimal time to administer glucarpidase is within 48–60 h from the start of the HDMTX infusion to prevent life‐threatening toxicities [47].

## Author Contributions

**Zhongbo Hu:** conceptualization (lead), data curation (lead), formal analysis (equal), investigation (lead), methodology (lead), project administration (equal), resources (equal), validation (equal), writing – original draft (lead), writing – review and editing (lead). **Andrea M. Escalera‐Joy:** data curation (supporting), methodology (equal), writing – original draft (supporting), writing – review and editing (equal). **Emily Ashcraft:** data curation (equal), formal analysis (equal), methodology (equal), resources (equal), software (equal), validation (equal), writing – original draft (equal), writing – review and editing (equal). **Rushil Acharya:** project administration (equal), resources (equal), visualization (equal), writing – review and editing (equal). **Sima Jeha:** funding acquisition (equal), investigation (equal), resources (equal), supervision (equal). **Cheng Cheng:** conceptualization (equal), data curation (equal), formal analysis (equal), investigation (equal), methodology (equal), software (equal), supervision (equal), validation (equal), writing – review and editing (equal). **Ching‐Hon Pui:** funding acquisition (equal), resources (equal), supervision (equal), validation (equal), writing – review and editing (equal).

## Ethics Statement

The study was approved by St. Jude Children's Research Hospital Institutional Review Board. The original St. Jude Total Therapy Study 16's ClinicalTrials.gov Identifier: NCT00549848.

## Conflicts of Interest

C.‐H.P. has received honoraria from Novartis, Amgen, and UpToDate and is on the Scientific Advisory board of Adaptive Inc. The remaining authors declare no competing financial interests.

## Supporting information


Data S1.

## Data Availability

Corresponding author will provide data upon request.
