# Correction to “Clinical Risk Factors for High‐Dose Methotrexate‐Induced Oral Mucositis Following Individualized Dosing”

**DOI:** 10.1002/cam4.71478

**Published:** 2026-02-10

Hu, Z., Escalera‐Joy, A., Ashcraft, E., Acharya, R., Jeha, S., Cheng, C. and Pui, C.‐H. (2024), Clinical Risk Factors for High‐Dose Methotrexate‐Induced Oral Mucositis Following Individualized Dosing. Cancer Med, 13: e70351. https://doi.org/10.1002/cam4.70351.

In paragraph 1, section 2.2 | MTX Concentration Monitoring, the text “using the ARK diagnostics homogeneous enzyme immunoassay (ARK Diagnostics', Fremont, CA)” was incorrect. It should be changed to “using assay by a fluorescence polarization immunoassay technique with a TDx/FLx analyzer (Abbott Diagnostics, Chicago, Illinois, U.S.A.).” The referenced citation 20 should read “P. R. Oeltgen, W. A. Shank Jr, R. A. Blouin, T. Clark.,” “Clinical Evaluation of the Abbott TDx Fluorescence Polarization Immunoassay Analyzer,” “Therapeutic *Drug Monitoring* 6 no. 3 (1984): 360–367.”

We apologize for these errors.

