# Supplementary material for: Clinical Risk Factors for High‐Dose Methotrexate‐Induced Oral Mucositis Following Individualized Dosing
Source: Cancer Med. 2024 Nov 1;13(21):e70351. doi: 10.1002/cam4.70351 (PMC11529650; doi:10.1002/cam4.70351)
Supplement: Supplementary file 1 — Data S1. [file CAM4-13-e70351-s001.docx]

**Supplemental Table 1**. Clinical symptoms during HDMTX associated with oral mucositis. CNS, central nervous system. ANC, absolute neutrophil count. NA, none available. () shows in % inside the content of the table.

|  | **LR patients** | | | **SHR patients** | | |
| --- | --- | --- | --- | --- | --- | --- |
|  | **Case (N=38)** | **Control (N=38)** | **P** | **Case**  **(N=76)** | **Control**  **(N=76)** | **P** |
| **Skin side effect** | | | | | | |
| Present | 7(18.4) | 0(0) | NA | 16(21.1) | 4(5.3) | 0.0027 |
| Absent | 31(81.6) | 38(100) |  | 60(78.9) | 72(94.7) |  |
| **CNS side effect** | | | | | | |
| Present | 3(8.1) | 1(2.6) | 0.3173 | 3(3.9) | 3(4.2) | 1 |
| Absent | 34(91.9) | 37(97.4) |  | 73(96.1) | 69(95.8) |  |
| **Fever during the course** | | | | | | |
| Present | 16(42.1) | 3(7.9) | 0.0016 | 19(25.0) | 9(11.8) | 0.0124 |
| Absent | 22(57.9) | 35(92.1) |  | 57(75.0) | 67(88.2) |  |
| **Neutropenia during the course (ANC ≤ 500)** | | | | | | |
| Present | 25(65.8) | 11(28.9) | 0.006 | 41(53.9) | 24(31.6) | 0.0079 |
| Absent | 13(34.2) | 27(71.1) |  | 35(46.1) | 52(68.4) |  |
| **Neutropenic fever during the course (ANC ≤ 500)** | | | | | | |
| Present | 10(26.3) | 2(5.3) | 0.0209 | 15(19.7) | 2(2.6) | 0.0008 |
| Absent | 28(35.9) | 36(94.7) |  | 61(80.3) | 74(97.4) |  |

**Supplemental Table 2**. Analysis of the association of laboratory results with oral mucositis during HDMTX. ANC, absolute neutrophil count. AKI, acute kidney injury.

|  | **LR patients** | | | | **SHR patients** | | | |
| --- | --- | --- | --- | --- | --- | --- | --- | --- |
|  | **Case (N=38)** | | **Control (N=38)** | **P** | **Case (N=76)** | | **Control (N=76)** | **P** |
| **Average of ANC before initiating HDMTX [Mean (SD)], unit mm^-3^** | | | | | | | | |
|  | 1186.4(719.6) | | 1334.7(660.4) | 0.2844 | 1246.1(880.7) | | 1303.2(1009.9) | 0.9362 |
| **Lowest ANC during HDMTX course [Mean (SD)], unit mm^-3^** | | | | | | | | |
|  | 540.5(354.5) | | 1000.8(671.6) | 0.0007 | 613.8(508.0) | | 1025.0(735.8) | <.0001 |
|  |  |  |  |  |  |  |  |  |
| **Previous AKI # (%)** | | | | | | | | |
| Present | 8(21.1) | | 5(13.5) | 0.3657 | 16(21.1) | | 9(11.8) | 0.1444 |
| Absent | 30(78.9) | | 32(86.5) |  | 60(78.9) | | 67(88.2) |  |
| **AKI during HDMTX course (%)** | | | | | | | | |
| Present | 11(28.9) | | 1(2.6) | 0.0039 | 19(25.0) | | 8(10.5) | 0.0164 |
| Absent | 27(71.1) | | 37(97.4) |  | 57(75.0) | | 68(89.5) |  |
| **Previously delayed MTX clearance (%)** | | | | | | | | |
| Present | 3(15.0) | | 3(15.8) | 1 | 8(24.2) | | 8(26.7) | 0.7389 |
| Absent | 17(85.0) | | 16(84.2) |  | 25(75.8) | | 22(73.3) |  |
| **Delayed MTX clearance during the HDMTX course (%)** | | | | | | | | |
| Present | 14(36.8) | | 3(7.9) | 0.0045 | 30(40.0) | | 16(21.1) | 0.0133 |
| Absent | 24(63.2) | | 35(92.1) |  | 45(60.0) | | 60(78.9) |  |

**Supplemental Table 3**. Analysis of the association of MTX dosages and kinetics with oral mucositis during HDMTX. Values are shown as Mean (SD). Only 42-Hour MTX level is associated with HDMTX induced oral mucositis in both LR and SHR group. 66-Hr MTX level is not significantly associated with MTX-induced oral mucositis. N, the number of patients in both case and control.

|  | **LR patients** | | | | **SHR patients** | | | | |
| --- | --- | --- | --- | --- | --- | --- | --- | --- | --- |
|  | **N** | **Case** | **Control** | **P** | **N** | **Case** | **Control** | **P** | |
| **42-Hr MTX level(μmol/L)** | | | | | | | | | |
|  | 38 | 0.95(4.10) | 0.34(0.17) | 0.0115 | 76 | 0.99(1.14) | 0.67(0.79) | 0.0179 |  |
| **66-Hr MTX level(μmol/L)** | | | | | | | | | |
|  | 9 | 0.35(0.48) | 0.10(0.05) | 0.2617 | 32 | 0.22(0.19) | 0.21(0.31) | 0.2809 |  |
| **MTX dosage (mg/m2, except Down syndrome patient)** | | | | | | | | | |
|  | 38 | 2479.0  (129.8) | 2426.0  (251.9) | 0.3125 | 75 | 4662.1  (728.9) | 4671.2  (645.0) | 0.9055 |  |
| **MTX AUC (μM*hr, except Down syndrome patient)** | | | | | | | | | |
|  | 38 | 937.1(330.1) | 810.4(190.2) | 0.1035 | 75 | 1869.0  (466.1) | 1745.4  (418.3) | 0.8920 |  |
| **Accumulated LCV dose (mg/m^2^)** | | | | | | | | | |
|  | 37 | 210.9(362.3) | 34.6(10.1) | <0.0001 | 75 | 238.8  (652.5) | 81.3  (121.9) | <0.0001 |  |

**Supplemental Table 4. Analysis of the association of the m**edications during HDMTX with oral mucositis during HDMTX. Number shows with patient number and () shows in % inside the content of the table. 6MP, mercaptopurine. HDMTX, high-dose methotrexate. ANC, absolute neutrophil count. TMP/SMX, trimethoprim-sulfamethoxazole.

|  | **LR patients** | | | | | **SHR patients** | | |
| --- | --- | --- | --- | --- | --- | --- | --- | --- |
|  | **Case (N=38)** | **Control (N=38)** | | | **P** | **Case**  **(N=76)** | **Control**  **(N=76)** | **P** |
| **6MP dose during HDMTX course** | | | | | | | | |
| Full dose | 15(39.5) | | 21(55.3) | | 0.2351 | 38(50.0) | 41(53.9) | 0.0495 |
| Reduced dose | 19(50.0) | | 16(42.1) | |  | 26(34.2) | 29(38.2) |  |
| Dose on hold | 4(10.5) | | 1(2.6) | |  | 12(15.8) | 6(7.9) |  |
| **Anti-hypertensive medicine during HDMTX course** | | | | | | | | |
| Present | 1(2.6) | | | 0(0) | NA | 6(7.9) | 5(6.6) | 0.763 |
| Absent | 37(97.4) | | | 38(100) |  | 70(92.1) | 71(93.4) |  |
| **TMP/SMX during HDMTX course** | | | | | | | | |
| Yes | 35(92.1) | | | 37(97.4) | 0.3173 | 71(93.4) | 67(88.2) | 0.2850 |
| No | 3(7.9) | | | 1(2.6) |  | 5(6.6) | 9(11.8) |  |

**Supplemental Table 5.**  Analysis of the association of the molecular and cytogenetic diagnosis with oral mucositis during HDMTX. Number shows with patient number and () shows in % inside the content of the table. #, 6 T-ALL patients with KMT2A positive.

|  | **LR patients** | | | **SHR patients** | | |
| --- | --- | --- | --- | --- | --- | --- |
| Molecular | **Case (N=38)** | **Control (N=38)** | **P** | **Case (N=76)** | **Control (N=76)** | **P** |
| KMT2A | 0(0.0) | 0(0.0) | NA | 7(12.5) | 7(12.5) | 0.7613 |
| BCR::ABL1 | 0(0.0) | 0(0.0) |  | 3(5.4) | 4(7.1) |  |
| ETV6::RUNX1 | 22(57.9) | 14(36.8) |  | 2(3.6) | 7(12.5) |  |
| Hyperdiploid | 12(31.6) | 16(42.1) |  | 11(19.6) | 10(17.9) |  |
| Low hypodiploid | 0(0.0) | 0(0.0) |  | 1(1.8) | 3(5.4) |  |
| Other | 4(10.5) | 8(21.1) |  |  |  |  |
| Immunophenotype |  |  |  |  |  | 1 |
| T-ALL | 0(0.0) | 0(0.0) |  | 20(26.3) | 20(26.3) |  |
|  |  |  |  |  |  |  |

**Supplemental Table 6. Differences in mean of MTX pharmacokinetics and other parameters between patients with one and 2 or more than 2 episodes of mucositis during courses of HDMTX.**

| **Differences in mean MTX pharmacokinetics and other parameters between patients with one and 2 or more than 2 episodes of mucositis during HDMTX** | | | | | | | | | |
| --- | --- | --- | --- | --- | --- | --- | --- | --- | --- |
|  | **1 mucositis toxicity** | | |  | **2+ mucositis toxicities** | | | |  |
| **Variable** | **N** | **Median** | **Min** | **Max** | **N** | **Median** | **Min** | **Max** | **p-value** |
| **MTX 42hr** | 98 | 0.555 | 0.07 | 6.52 | 16 | 0.7075 | 0.14 | 3.49 | 0.1055 |
| **MTX 66hr** | 67 | 0.16 | 0.02 | 2.05 | 14 | 0.2191667 | 0.045 | 0.915 | 0.3356 |
| **Absolute neutrophil count** | 98 | 1000 | 200 | 5300 | 16 | 1250 | 500 | 5200 | 0.2584 |
| **MTX dose** | 98 | 4260.5 | 500 | 6557 | 16 | 4453.75 | 2500 | 5400 | 0.817 |
| **MTX AUC** | 98 | 1504.85 | 195 | 3175.3 | 16 | 1762.81 | 1002.4 | 2524.5 | 0.0829 |
| **Accumulated LCV dose** | 97 | 65 | 30 | 1550 | 16 | 159.375 | 40 | 2835 | **0.017** |
| **Lowest ANC** | 98 | 495 | 0 | 2900 | 16 | 587.5 | 150 | 2200 | 0.1026 |

**Supplemental Figure 1. Accumulated LCV dose is significantly associated with HDMTX induced oral mucositis. But MTX dosage and AUC are not.**

*****, *p*<0.001. A. MTX dose. B MTX AUC. C. Accumulated leucovorin doses.**
